# Therapeutic effects of medicinal plants and their constituents on lung cancer, in vitro, in vivo and clinical evidence

**DOI:** 10.1111/jcmm.17936

**Published:** 2023-09-12

**Authors:** Arghavan Memarzia, Saeideh Saadat, Fereshteh Asgharzadeh, Sepide Behrouz, Gert Folkerts, Mohammad Hossein Boskabady

**Affiliations:** ^1^ Applied Biomedical Research Center Mashhad University of Medical Sciences Mashhad Iran; ^2^ Department of Physiology, Faculty of Medicine Mashhad University of Medical Sciences Mashhad Iran; ^3^ Department of Physiology, School of Medicine Zahedan University of Medical Sciences Zahedan Iran; ^4^ Department of Animal Science, Faculty of Agriculture University of Birjand Birjand Iran; ^5^ Division of Pharmacology, Utrecht Institute for Pharmaceutical Sciences (UIPS), Faculty of Science Utrecht University Utrecht Netherlands

**Keywords:** apoptosis, in vitro, in vivo, lung cancer, medical plants, metastasis

## Abstract

The most common type of cancer in the world is lung cancer. Traditional treatments have an important role in cancer therapy. In the present review, the most recent findings on the effects of medicinal plants and their constituents or natural products (NP) in treating lung cancer are discussed. Empirical studies until the end of March 2022 were searched using the appropriate keywords through the databases PubMed, Science Direct and Scopus. The extracts and essential oils tested were all shown to effect lung cancer by several mechanisms including decreased tumour weight and volume, cell viability and modulation of cytokine. Some plant constituents increased expression of apoptotic proteins, the proportion of cells in the G2/M phase and subG0/G1 phase, and Cyt c levels. Also, natural products (NP) activate apoptotic pathways in lung cancer cell including p‐JNK, Akt/mTOR, PI3/ AKT\ and Bax, Bcl2, but suppressed AXL phosphorylation. Plant‐derived substances altered the cell morphology, reduced cell migration and metastasis, oxidative marker production, p‐eIF2α and GRP78, IgG, IgM levels and reduced leukocyte counts, LDH, GGT, 5′NT and carcinoembryonic antigen (CEA). Therefore, medicinal plant extracts and their constituents could have promising therapeutic value for lung cancer, especially if used in combination with ordinary anti‐cancer drugs.

## 1 INTRODUCTION

It is estimated that 70% of cancer cases in the world are occurring in low‐ and middle‐income countries and, as with many cancers, early diagnosis increases the survival rate by up to 20%.^1^, ^2^ Tobacco smoke accounts for more than 80% of lung cancer cases, making it the most preventable cancer of this century.^3^, ^4^, ^5^ However, familial history, genetics and biological gender also pose effective risk factors. It was reported that lung cancer is more prevalent in women that smoke or who are exposed to cigarette or tobacco smoke, and a lifetime of smoking increases the risk of developing lung cancer by 20‐ to 30‐fold.^6^ Non‐invasive screening approaches such as chest imaging,^7^ monitoring of biomarkers: proteins,^8^, ^9^, ^10^ auto‐antibodies, early CDT‐Lung test,^11^ gene expression profiles^12^ and microRNAs^13^ in the blood or airway epithelium, evaluating mutations or methylation in circulating tumour DNA (ctDNA),^14^, ^15^, ^16^, ^17^, ^18^ as well as learning the history of chronic exposure to smoke and autoimmune diseases^13^ are helpful for early diagnosis of lung cancer. The two main types of lung cancer are small cell lung cancer (SCLC) and non‐small cell lung cancer (NSCLC). SCLC occurs mainly in the central airways including bronchioloalveolar cells, whereas NSCLC types originate in the neuroendocrine system.^4^ Radiotherapy, chemotherapy and surgery are common treatments, depending on the stage of the cancer. For example, in the early stages of cancer surgery is best, and in advanced stages, a combination of radiotherapy and surgery are appropriate treatment methods. Cisplatin or carboplatin‐based combination therapies are standard treatments of metastatic lung cancer.^19^

Alternative treatments in complement with traditional methods have been shown to be helpful in the treatment of various types of cancer, and more recently, medicinal plants^20^, ^21^, ^22^ are reported as effective alternative treatments of lung cancer. Their anti‐cancer properties include cytotoxicity (being toxic to cancer cells), inducing cancer cell apoptosis, inhibiting tumour angiogenesis and inhibiting cancer cell proliferation and growth. These impacts on cancer cells by medicinal plants and their derivatives occur through a variety of cellulare pathways and molecular processes, including: altering reactive oxygen species (ROS) generation, modulating transcriptional activators, causing mitochondrial membrane potential loss by P53 (promoter of apoptosis), inhibiting the proliferation of the transformed cell by CD95, Cyt.c and Smac release, down‐regulating Nuclear factor kappa B (NFk‐B), activator protein 1 (AP‐1), early growth response protein 1 (EGR‐1), cyclooxygenase 2 (COX‐2), lipoxygenase (LOX), matrix metallopeptidase 9 (MMP‐9), tumour necrosis factor (TNF), chemokine, epidermal growth factor receptor (EGFR), human epidermal growth factor receptor 2 (HER2) and inhibiting c‐Jun N‐terminal kinases (JNK) and serine/threonine kinase (STK4) pathway. Furthermore, medicinal plant extracts have been found to inhibit metastasis in various tumour cancers (breast, hepatic, colon and stomach cancer) by reducing MMP‐2 release of cytochrome c (Cyt c), activating Cas‐3 and ‐9, down‐regulating anti‐apoptotic proteins Bcl‐XL and integrin associated proteins and altering the ratio of Bcl2 family proteins.^23^

In the present article, we discuss the 11 medicinal plants and their components or natural products (NP) that have proven most effective against lung cancer. We collate findings from in vitro and in vivo studies and clinical trials and provide the proposed molecular and cellular mechanisms of action. The biochemical properties of each plant and its constituents are described before exploring their potential as an alternative and complementary treatment of lung cancer.

## 2 METHODS

In vivo and in vitro reports regarding the effect of NP on lung cancer were explored using the databases PubMed, Science Direct and Scopus until the end of March 2022. Accordingly, keywords including lung cancer, medicinal plants, plant constituents, NP, in vivo, in vitro and clinical studies were used.

**FIGURE 1:**
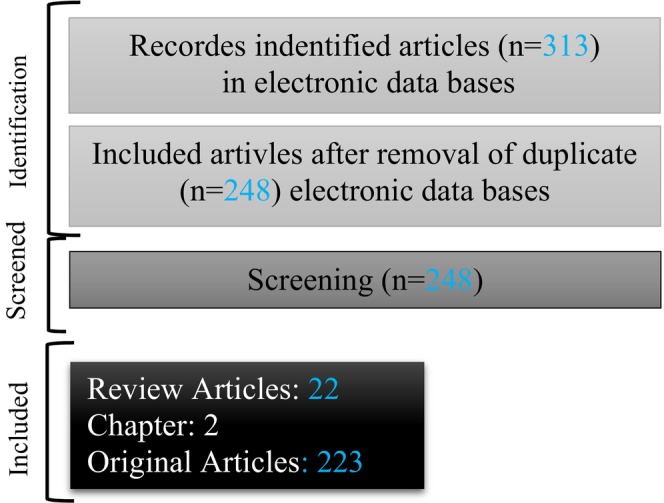
Flowchart of the selecting studies in the current review.

Several criteria were used to select eligible studies for this review: (1) in vitro, in vivo (animal) and human studies, (2) NP supplementation and their dosage, (3) the effect of NP on lung cancer, (5) English language articles published in well‐known international journals. The reference classification is depicted in Figure 1.

## 3 RESULTS

### 3.1 The effect of various medicinal herbs and their constituents on lung cancer

#### 3.1.1 Zataria multiflora

*Zataria multiflora* (*Z. multiflora*) is a traditional herb belonging to the Lamiaceae family and grows in the southern and central regions of Iran. Application of *Z. multiflora* extract, and its constituent carvacrol, led to various pharmacological effects including anti‐inflammation, immunomodulation and antioxidation. The plant extract and carvacrol also reduced paraquat (herbicide)‐induced lung injury via activation of PPAR‐γ.^24^, ^25^, ^26^, ^27^, ^28^, ^29^, ^30^, ^31^, ^32^, ^33^ Anti‐cancer effects of *Z. multiflora* and carvacrol have been reported in several studies that we discuss below.

Regarding the safety and efficacy of *Z. multiflora*, the results of two animal studies indicated that, due to phenol components including carvacrol and thymol, reduction of ROS levels in tissue but in doses more than 0.4 mL/kg for 14 days, *Z. multiflora* reduced the safety but increased toxicity.^34^, ^35^ However, in healthy human carvacrol at doses of 1 and 2 mg/kg/day for 1 month did not show any side effects.^36^ The effects of *Z. multiflora* extract and carvacrol on lung cancer are summarized in Table 1.

**TABLE 1:** The effects of *Zataria multiflora* and *Nigella sativa* and their derivatives, carvacrol and thymoquinone, on lung cancer.

Study type	Type of lung cancer	Preparations	Dose	Effects	Ref.
	A549 cell	CN	0–150 μg/mL	Decreased MAPK p38, ERK activation, COX‐2 and VEGF expression	^39^
	NSCLC	Carvacrol	0, 30, 100 and 300 μM	Decreased AXL protein expiration, inhibited AXL phosphorylation	^44^
	NSCLC	Carvacrol nanoemulsion	25, 50 and 100 μg/mL	Decreased ROS, regulated p‐JNK, Bax, Bcl2, increased Cyt c, caspase, apoptosis, down‐regulated CHOP, p‐eIF2α, GRP78 expression	^43^
	NSCLC	Carvacrol	100–1000 μM	Decreased morphology changes, cell growth	^42^
	Doxorubicin resistant‐A549 cell line	Carvacrol nanoemulsion	5, 25 and 50 μg/mL after 24 h	Elevated Bax, Cyt c, cleaved caspase 3 and 9, p21 protein expression, reduced CDK2, CDK4, CDK6, Cyclin E, cyclin D1	^43^
	NCI‐H460 cells	TQ	1.25, 0.5, 5 μM, for 24, 48 and 72 hrs	Reduced cell proliferation, cytokines expression, ENA‐78, GRO‐alpha, increased apoptosis level, cell viability	^47^
	NSCLC	TQ	10, 20, 40 μmol/L	Inhibited cell proliferation, migration PCNA, cyclin D1, MMP2, MMP9 mRNA, activation ERK1/2 pathway, cell cycle	^55^
	A549 non‐small cell	TQ	2, 5, 10 μM	Up‐regulated Bax, p21, receptor 1 and 2 expressions, Bax/Bcl2 ratio, down‐regulated Bcl2 proteins, inflammatory markers, ROS, cyclin D, NF‐kappa B and IKK1 expression	^56^
In vivo	Tumour‐bearing BALB/c mice	*Z. multiflora* Essential oil	500 mg/kg	Increased TNF‐α, IFN‐γ, IL‐2, decreased IL‐4 level	^38^
	Lung cancer induced‐B(a)P	Carvacrol	25 and 50 mg/kg, 7 days	Enhanced antioxidants, iNOS, NF‐κB and COX‐2 expressions, decreased positive stained cells	^40^
	Lung cancer model (athymic nude mice)	Carvacrol	50 and 100 mg/kg	Reduced tumour growth and weight, increased p‐JNK, Bax, Bcl2, Cyt C, caspase‐3, caspase‐9 and β‐ Actin expression	^43^
	Rat multi‐organ carcinogenesis	*N. sativa* volatile oil	1000 or 4000 ppm, 30 weeks	Reduced carcinogenesis incidences, multiplicities	^54^
	Lung cancer model of mice	TQ	5, 20 mg/kg	Decreased tumour volume and weight, NF‐KB	^47^
	CD1‐nude mice	TQ	5, 10, 10 mg/kg	Reduced p‐AKT, p‐mTOR, caspase‐3, p‐53, NF‐κB expression	^58^
	B(a)P‐induced rat's lung cancer	TQ	50 mg/kg b.w.	Decreased NFk‐B expression, ROS, MDA, NO, increased apoptosis, CAT and SOD activities	^62^

Abbreviations: B(a)P, benzo(a)pyrene; CN, carvacrol nanoemulsion; IFN‐γ, interferon‐gamma, IL‐2, interleukin‐2; NF‐κB, nuclear factor kappa B; NSCLC, non‐small cell lung cancer; NSE, seed extract; Ref, references; ROS, reactive oxygen species; SPBP, soy‐phospholipid‐based phytosomes; TNF alpha, tumour necrosis factor‐alpha; TQ, thymoquinone; VEGF, vascular endothelial growth factor.

##### Extract preparation

For the preparation of methanolic extract of *Z. multiflora* its dried powder was mixed with 85% methanol for 48 h and filtered and the ethanol was removed by rotary at 40C°.^37^ Also, *Z. multiflora* essential oil was prepared from the air‐dried plants through hydrodistillation for 3 h using an all‐glass Clevenger‐type apparatus. The essential oil was dehydrated over anhydrous sodium sulphate and stored at 4°C.^38^

##### Growth‐inhibitory activity in cell culture

Treatment with *Z. multiflora* methanolic extract down‐regulated c‐MYC levels and up‐regulated p53 in the U266 multiple myeloma cell line.^37^ Pre‐treatment A549 (human lung carcinoma), HCT‐116 (human colorectal carcinoma), HepG2 (human hepatocellular carcinoma) and B16F10 (mouse melanoma) cells with carvacrol (25 and 50 mg/kg for 7 days, orally) before inducing benzo(a)pyrene [B(a)P] enhanced antioxidant enzyme activity.^39^ Furthermore, carvacrol pre‐treatment down‐regulated protein expression of inducible nitric oxide synthase (iNOS), NF‐κB and COX‐2 causing a decrease in the number of cancer marker cells and restoring histopathological changes made to the lung tissues.^40^ Administration of carvacrol nanoemulsion (CANE, 0–150 μg/mL) decreased MAPK p38 and ERK activation as well as COX‐2 and vascular endothelial growth factor (VEGF) expression.^39^ CN can also bind to COX‐2 and VEGF and activate allosteric sites of CD31 with low binding energy in A549 (adenocarcinoma cell line) cells.^39^ Another study found that carvacrol suppressed the viability of NCI‐H1299 cells by inducing apoptosis by inhibiting protein and mRNA expression levels of Cas‐9 and elevating the expression of MMP‐9 and TIMP‐1.^41^ Treatment with carvacrol (100, 250, 500 and 1000 μM) for 24 h caused morphological changes in NSCLC (A549) cells by improving cell rounding, causing cytoplasmic blebbing and irregularity in shape, leading to fewer cells and inhibiting rates of cell growth.^42^ Carvacrol nanoemulsion (25, 50 and 100 μg/mL) also decreased ROS and regulated apoptosis via p‐JNK, Bax and Bcl2 and increased Cyt c and activated Cas cascades in human lung adenocarcinoma A549 cells. Following administration of carvacrol, an MTT assay revealed increased A549 cell viability and further testing found increases in ROS production, a decrease in Ca2+ release, down‐regulation of CHOP, p‐eIF2α, GRP78 and apoptotic markers.^43^ Western blots and reverse transcription polymerase chain reaction (RT‐PCR) tests found that 24 h administration of carvacrol inhibited the expression of AXL proteinase and suppressed AXL phosphorylation upon stimulation of neuronal NSCLC cells with ligands.^44^ In addition, carvacrol treatment led to inhibited cell proliferation and migration of the NSCLC cells.^44^ Finally, CN treatment was found to reduce the number of cell proliferation enzymes: cyclin‐dependent kinase 2 (CDK2), CDK4, CDK6, Cyclin E, Cyclin D1 and enhanced expression of p21 protein in the doxorubicin resistant‐A549 cell line.^45^

##### Anti‐tumour activity in vivo

*Z. multiflora* essential oil (500 mg/kg) reduced tumour weight and balanced T helper 1 levels by increasing the secretion of TNF‐α, Interferon‐gamma (IFN‐γ) and IL‐2 and decreasing IL‐4 levels. However, no significant effects on aminotransferase and alanine aminotransferase activity were found.^38^

Two doses of carvacrol nanoemulsion (CANE, and 100 mg/Kg) over 4 weeks in an athymic nude mouse model significantly reduced tumour growth and tumour weight by 34.2% and 62.1%, respectively, and increased the expression of p‐JNK, Bax, Bcl2, Cyt C, Cas‐3, Cas‐9 and β‐actin in lung tissue.^43^ In vivo indicated that CN treatment (50,100 mg/kg) decreased cell growth and levels of MMP, decreased the activation of MAPK p38 and ERK, decreased the expression of VEGF and CD31 and decreased anti‐angiogenesis effects.^39^

Overall, treatment with *Z. multiflora* extract, and its main constituent carvacrol, reduced lung cancer virality by decreasing tumour weight and volume, increasing the expression of apoptotic proteins, modulating cytokine levels and cancer cell viability, as well as regulating specific pathways and cancer tumour cell numbers. Therefore, *Z. multiflora* extract and carvacrol are worth testing as a part of contemporary treatment strategies for lung cancer.

#### 3.1.2 Nigella sativa

A variety of diseases have been treated with *Nigella sativa* (*N. sativa*) seeds for thousands of years across the African and Asian continents. For example, in the south‐west of Asia, this plant has been used both as a food additive and herbal medicine.^46^ A powerful anti‐cancer compound extracted from *N. sativa* seeds is thymoquinone (TQ).^47^ Several studies have found both *N. sativa* and TQ to have anti‐inflammatory, anti‐oxidative and immunomodulatory properties, and are also effective treatments against allergic disorders, lung cancer and tumour morphology.^48^, ^49^, ^50^, ^51^

Regarding the safety and toxicity of black seeds and TQ, dependent on the type of animal model and administration manner, the results demonstrated that the toxicity effects of oral administration were lower than intraperitoneal injection and administration of more than 2 and 3 g/kg in the mice increased the risk of the organ toxicity of *N. sativa* extract and TQ.^52^ The results of a phase I clinical trial to evaluate the safety of thymoquinone‐rich black cumin oil (BlaQmax®) on healthy subjects consuming black cumin oil formulation containing 5% at a dose of 200 mg/adult/day for 90 days showed a well‐safe profile of the plant.^53^ As shown in Table 1, *N. sativa* and TQ are both potential treatments for lung cancer.

##### Extract preparation

Cleaned and dried seeds of *N. sativa* were extracted with 95% aqueous methanol for 24 h. The extract was filtered by a Buchner funnel and re‐extracted with 0% methanol for an additional 2 h and methanol was removed with a rotatory evaporator at 40°C.^49^ *N. sativa* volatile oil used in Salim's study was purchased from Kahira Pharm. and Chem. Co., Cairo, Egypt.^54^

##### Growth‐inhibitory activity in cell culture

The administration of essential oil *N. sativa* gold nanoparticles (NsEO‐AuNPs) decreased the hydrophobicity index causing significant suppression of *S. aureus* (78%) and *V. harveyi* (46%) biofilm formation, which are common infections in patients with lung cancer.^54^ A study using *N. sativa* plant seed extract (NSE) treatment found it caused a significant down‐regulation of cancer cell viability and altered their morphology. Similarly, NSE treatment (0.01, 0.025, 0.05, 0.1, 0.25, 0.50, 1 mg/mL for 24 h) on human lung cancer cells significantly reduced their viability.^49^ TQ treatment (1.25, 2.5 and 5 μM, for 24, 48 and 72 h) of NCI‐H460 cells (NSCLC cell line) reduced cell proliferation, expression of cytokines, epithelial‐neutrophil activating peptide (ENA‐78) and GRO‐alpha and increased apoptosis occurrence and reduced cell viability.^47^ Administration of TQ (10, 20, 40 μmol/L) on a SCLC cell line also inhibited cell proliferation, migration, proliferating cell nuclear antigen (PCNA), cyclin D1, MMP2 and MMP9 mRNA levels, activated the extracellular signal‐regulated kinase 1/2) ERK1/2 (pathway and inhibited cell cycle via P16 expression and the gelatinase activities of MMP2 and MMP9.^55^ Treatment with TQ (2, 5, 10 μM) on NCLC cells exposed to B(a)P up‐regulated Bax and p21 levels, increased receptor 1 and 2 expression and the Bax/Bcl2 ratio, whilst down‐regulating Bcl2 proteins, inflammatory markers, ROS, cyclin D, NF‐kappa B and IKK1 expression.^56^ Treatment with TQ‐loaded soy‐phospholipid‐based phytosomes (0.5, 1.0, 2.0, 4.0, 6.0, 8.0 and 12.0 h) was found to activate Cas‐3 and reduce oxidative stress markers in A549 cells using the annexin V staining technique.^57^ Combined application of indirubin‐3‐monoxime and TQ on A549 cancer cells did increase apoptosis markers and also reduced the Bcl‐2/Bax ratio, inhibiting migration and metastasis of the cancer cells.^58^ Administration of TQ also increased natural killer (NK) cell tumoricidal activity and enhanced IFN‐γ secretion via NK cells and NK cell‐mediated killing of NSCLC cells.^59^ In addition, inhibition of GTPase KRas, Sir‐2, ALK5 and β‐catenin were shown when TQ was applied to lung cancer cells.^60^

##### Anti‐tumour activity in vivo

The use of honey and *N. sativa* (0.2 g *N. sativa* or 5 g honey/day) as supplements in Sprague Dawley rats showed protection against oxidative carcinogenesis induced by methyl nitrosourea in lung, skin and colon tissues.^61^ Oral administration of *N. sativa* volatile oil (1000 or 4000 ppm) for 30 weeks to male Wistar rats reduced the size of some tumours, including in the lungs.^54^ Similarly, administration of TQ (5 and 20 mg/kg) decreased the size and weight of tumours and NF‐KB in an animal lung cancer xenograft model.^47^ Treatment of a mouse lung cancer model with indirubin‐3‐monoxime and TQ (5, 10, 10 mg/kg) significantly reduced the expression of Phospho‐Akt (p‐AKT), Phospho‐Mammalian target of rapamycin (p‐mTOR), Cas‐3, p‐53 and NF‐κB.^58^ Finally, administration of TQ (50 mg/kg) decreased NFk‐B expression, ROS, MDA and NO levels and increased apoptosis and CAT and SOD activity in B(a)P‐induced lung cancer in rats.^62^

The above in vitro and in vivo results indicate that *N. sativa* and its constituent TQ could be promising candidates for treating lung cancer. Their therapeutic actions involve decreasing cancer cell viability and altering their morphology and levels of Bcl2 proteins, inflammatory markers, oxidative stress parameters and NF‐kappa whilst increasing Bax/Bcl2 ratio and levels of apoptosis.

#### 3.1.3 Crocus sativus

*Crocus sativus L* (*C. sativus*) is mainly cultivated in Iran and is a member of the Iridaceae family. Chemical analyses of *C. sativus* extract indicate that the most critical constituents are carotenoids, crocin, crocetin and the monoterpene aldehydes crocin and safranal.^63^ Studies have reported that *C. sativus* and its critical constituents decrease white blood cell (WBC) counts, reduce oxidative stress markers, impact airway responsiveness and also have immunomodulatory effects.^64^, ^65^, ^66^

Regarding the safety of *C. sativus*, its oral administration of 200 and 400 mg/day for 7 days, indicated the changes in some haematological and biochemical parameters but, these changes were in normal ranges which were not important clinically.^67^ The effect of *C. sativus* and its constituents on lung cancer and their potential mechanism was also reported in numerous studies (Table 2).

**TABLE 2:** The effects of *Crocus sativus*, *Ocimum basilicum*, *Ferula persica* and *Ferula szowitsiana* and their constituents on lung cancer.

Study type	Type of lung cancer	Preparations	Dose	Effects	Ref.
In vitro	L929 cell line	Saffron ethanolic extract	500–2000 μg/mL, 24 and 48 h	Reduced cell number and viability	^68^
	GCL‐induced A549 cells	Crocin	500 μM, 48 h	Reduced GSHand ROS, enhanced GCL expression, activated Nrf2	^72^
	A549 cell	Linalool	0–2.0 mM	Inhibited cell proliferation, migration, increased G0/G1 cell cycle arrest, anti‐metastatic	^81^
	A549 cell	*F. persica* methanolic extract	400 μg/mL, 48 and 72 h	Up‐regulated P53, Bax and caspase‐9, activated multiple apoptotic pathways	^86^
	A549 cell	*F. persica* methanolic extract	100 μg/mL	Decreased cytotoxicity and cell proliferation	^85^
In vivo	Mice lung cancer model		100 mg/kg, for 28 days	Decreased xenograft tumour size and reduced Cas‐3, −8 and − 9 expression	^70^

Abbreviations: Bax, B‐cell lymphoma 2‐associated X protein; Bcl‐2, B‐cell lymphoma 2; Ref, references; and ROS, reactive oxygen species.

##### Extract preparation

For the preparation of ethanolic extract, 1 g of the dried saffron stigma was extracted with 10 mL ethanol (96%) for 2 h in an ultrasonic bath. Then the extract was filtered and concentrated in a vacuum evaporator.^68^

##### Growth‐inhibitory activity in cell culture

Treatment with ethanolic saffron extract (500, 1000, 1500 and 2000 μg/mL for 24 and 48 h) reduced cell number and viability of L929 lung cancer cells.^68^ The increased apoptotic level was indicated using annexin V‐fluorescein isothiocyanate in A549 cancer cells that had been treated with *C. sativus* extract.^69^ Furthermore, administration of saffron extract (100 mg/kg for 28 days) decreased xenograft tumour size and reduced Cas‐3, −8 and −9 expression.^70^

Treatment of lung cancer cells with crocin (1, 2, 4, 8 and 16 mg/mL) markedly enhanced the mRNA levels of p53 and Bax and significantly down‐regulated Bcl‐2 mRNA expression.^71^ Another study using crocin treatment (500 μM for 48 h) on glutamate‐cysteine ligase (GCL)‐stimulated A549 cancer cells found it reduced GSH levels by enhancing GCL expression via activation of Nrf2, but inhibited the release of ROS in.^72^

##### Anti‐tumour activity in vivo

Albino mice with B(a)P‐induced lung cancer were treated with crocetin (50 mg/kg) and cancer cell proliferation was reduced by 68% and 45% in weeks 8 and 18 respectively, and glycoproteins and polyamines were significantly altered.^71^

The above results suggest that *C. sativus*, crocin and crocetin down‐regulate mRNA levels of major proteins of apoptosis in lung cancer cells. Therefore, *C. sativus*, crocin and crocetin could be considered as treatment strategies for lung cancer.

#### 3.1.4 Ocimum basilicum

*Ocimum basilicum L*. (*O. basilicum*), or basil, is the main part of the oil crop used in traditional medicine that belongs to the Lamiaceae family. It is widely found in regions of central and southeast Asia, including Iran and Pakistan.^48^, ^73^ Previous studies indicated that linalool is the medicinal constituent of *O. basilicum*.^74^ The anti‐cancer effects of *O. basilicum* and its constituents have been demonstrated by Alkhateeb et al.^75^ Experimental and clinical studies found *O. basilicum* and its derivatives reduced inflammation and oxidative stress and improved immune function in patients with asthma, COPD, lung cancer and allergy diseases.^76^, ^77^, ^78^

Safety results demonstrated that acute effects of *O. basilicum* were shown in the LD50 > 5 mg/kg such as haematocrit, platelets and RBC decreasing and oral toxicity in patients.^79^ It was shown that *O. basilicum* consumption in food and drug preparations is safe. However, a concentration limit for alkylbenzenes should be considered, and the plant chemotypes with equal or lower levels of these alkylbenzenes should be used.^80^ The effects of *O. basilicum* on lung tumours are summarized in Table 2.

##### Growth‐inhibitory activity in cell culture

Treatment of A549 cancer cells with linalool (0–2.0 mM) inhibited cell proliferation and migration but increased the G0/G1 cell cycle arrest. Linalool treatment also sensitized cancer cells and anti‐metastatic markers were recorded in the treated A549 cells.^81^ Finally, linalool significantly decreased cancer cell viability even if only administered for 4 h.^81^ The above in vitro studies support the hypothesis that *O. basilicum* and its constituent linalool are a natural therapeutic against lung cancer cells and support further research to test the hypothesis.

#### 3.1.5 *Ferula assa‐foetida* and *F. Szowitsiana*

The genus *Ferula* are perennial flowering plants belonging to the Apiaceae (Umbelliferae) family. *Ferula* growth spans the eastern Mediterranean regions all the way to central Asia.^82^ This genus consists of about 170 species of which 30 are found in Iran. The anti‐tumour and cytotoxic effects against multifarious cancer cell lines, anti‐inflammatory, antioxidant, immunomodulatory and anti‐bacterial activities, reduced lipid peroxidation and down‐regulation of matrix metalloprotease expression caused by *F. szowitsiana* and its constituents have been reported in several studies (75–82). As well as *F. szowitsiana*, studies have found *F. persica* and its derivative coumarin may possess therapeutic properties.

Administration *F. persica* known as food additive until 1.5–2 mg/kg incresed the fibroblast cells in male patients and improved libido but did not report serious or acute side effects.^83^, ^84^ The effects of *F. persica* and coumarin on lung cancer are summarized in Table 2.

##### Extract preparation

Total plant extracts were obtained by extraction of dried and milled aerial parts of the plants with 80% methanol (1:10) using the maceration method for 4 days. After every 24 h, the mixture was filtered and a new solvent was added to the plant powder. The combined extracts were concentrated to dryness under vacuum pressure.^85^ In the other study, the extract of the air‐dried roots of *F. szowitsiana* (100 g) was prepared using the Soxhlet‐extracted method with dichloromethane and methanol (1 L of each) and then was dried by evaporator.^86^

##### Growth‐inhibitory activity in cell culture

Treatment of A549 cells with *F. szowitsiana* extract (400 μg/mL for 48 and 72 h) altered anti‐apoptosis, pro‐apoptosis and tumour suppressor gene expression, up‐regulated P53, Bax and Cas‐9 and also activated multiple apoptotic pathways and antiproliferation activity.^86^ Treatment with *F. persica* extract (100 μg/mL) decreased cell viability and cell proliferation in MDBK, A549, HT29, HepG2 and MCF7 cell lines.^85^

Cell proliferation, invasion, metastasis and autophagy were reduced in lung cancer cells treated with terpenoids. The inhibitory effects of terpenoids on cyclins and CDK were shown.^81^ Triterpenoids (25 mg/kg) inhibited the ROS accumulation via cytotoxicity of cisplatin by blocking autophagic flux.^87^ Human lung cancer cells treated with *F. szowitsiana* extract showed decreased cytotoxicity via SK‐LU‐1, SPC‐A‐1 and 95D tests and were also less viable.^88^ Inhibition of cell growth via increasing G1 cell cycle arrest, morphological cells and annexin were reported in NSCLC cell lines treated with coumarin (10–160 μg/m).^89^ The level of ROS and glutathione (GSH) in human alveolar epithelial A549 cancer cells was decreased and thiobarbituric acid reactive substance (TBARS) was markedly increased when they were treated with coumarin.^90^ Coumarin derivatives (12 μM) significantly decreased the migration of IL‐1β‐stimulated cells and IL‐1β levels and inhibited F‐actin reorganization in A549 cell lines.^91^ An earlier study found that administration of coumarin‐based benzopyranone derivatives (20 μM) up‐regulated the apoptotic pathway via Bax protein expression but down‐regulated Bcl‐2 protein expression in human lung (A549) cancer cells.^92^

Based on the above findings, further investigation in *F. persica* and its main constituent coumarin as a possible treatment for lung cancer via the inhibition of cyclins and CDK, their reducing effects on cancer cell migration and metastasis mediated by cytokines such as IL‐1β, oxidative marker production, cell viability and cytotoxicity warrant further investigation.

#### 3.1.6 Curcuma longa

*Turmeric*, also known as *Curcuma longa* (*C. longa*), is a part of the Zingiberaceae family found in some regions of Asia, being particularly abundant in India. In addition to its multiple health benefits, *C. longa* is an important ingredient in recipes.^93^, ^94^ Previous studies demonstrate various pharmacological properties of *C. longa* and curcumin (CUR) including: antioxidant (7), anti‐inflammatory,^95^ anti‐tumour,^96^ anti‐tussive,^97^ anti‐diabetic,^98^ anti‐convulsant^99^ and hepatoprotective effects.^100^ The anti‐inflammatory, antioxidant and immunomodulatory effects of CUR have also been reported in experimental lung disorders.^101^ Some studies have looked at the potential anti‐tumour mechanisms that *C. longa* and CUR adopt but further investigation is still needed to ascertain how they work.

Clinical results indicated that administration of *C. longa* and curcumin for 3 months, up to 8000 mg/d did not show side effects in patients, thus, it was recommended that using 2500 mg of *C. longa* was safe in humans.^102^ Table 3 summarizes the effects of *C. longa* and CUR on lung cancer.

**TABLE 3:** The effects of *Curcuma longa* and *Achillea millefolium*and their constituents on lung cancer.

Study type	Type of lung cancer	Preparations	Dose	Effects	Ref.
In vitro	A549 and H460 cells	CURoid extract	0.2%–2%, 8 days	Increased caspase‐3, caspase‐8 and caspase‐9 activities and Cyt c, CDK1 and cyclin B expression	^104^
	Lung cancer in vitro model	N‐hexane extract of *C. longa*	IC50, 0.23–0.28 mg/mL, 24	Inhibited telomerase‐ effect on cell line A549	^103^
	Lung cancer stem cells	CUR	5–40 μM, 7 days	Inhibited Wnt/β‐catenin, Sonic Hedgehog pathways	^106^
	A549 cells	CUR	25–100 μM, 6–48 h	Down‐regulated UCA1, inhibited LIN‐28A through the activation of miRNA‐98	^108^
	A549 cell	Nanoencapsulated CUR‐Fe3O4	0–120 μM, 24, 48 and 72 h	Reduced proliferation and hTERT gene expression	^109^
	Lung cancer cells	CUR	160 μM, 12–72 h	Decreased p53, BCL‐2, BCL‐XL, Bak and Caspase genes expression	^111^
	Lung cancer cells	CUR	10 μM	Regulated axon guidance, glioma, ErbB tyrosine kinase receptor signalling pathways	^112^
	Human NCI‐H292 LSCC cell line	CUR	40 μM, 4 h	Inhibited human NCI‐H292 cells growth, increased FOXA2 expression	^113^
	H460 cells	CUR	20 and 40 μM	Inhibited JAK2 activity, reduced tumour spheres by inhibiting JAK2/STAT3 pathway	^115^
	NSCLC patients	CUR	1 g/kg, once daily	Enhanced efficacy of EGFR‐TKIs and overcome the EGFR‐TKI resistance	^116^
	A549 cells	CUR	6 μM, 48 h	Suppressed EZH2 but reduced NOTCH1 expression via the EZH2 suppression	^124^
	Lung cancer cells	CUR	15, 30, 45 and 60 μmol/L	Suppressed GLUT1, MT1‐MMP, MMP2 expressions, attenuated GLUT1/MT1‐MMP/MMP2 pathway	^125^
	NSCLC cells	CUR	10, 20 and 30 μM, 2 h	Decreased MTP, ROS, activated DNA‐ DRP, mitochondrial apoptosis	^127^
	A549 cells	CUR derivative MHMM‐41	8 μM and 16 μM for 12 h	Induced ROS‐mediated apoptosis, blocked migration	^128^
	NCI‐H460 cells	CUR	30 μmol/L	Inhibited cell migration, tube formation, transfected pMXs‐Stat3C	^129^
	A549 cells	Kaempferol	17.5, 35, 52.5, 70 μM	Inhibited Akt‐1 phosphorylation, MEK1/2, activated MAPK, induced cell apoptosis	^142^
	A549 cells	Kaempferol	10, 25, 50 μM	Down‐regulated Smad3PCPC formation with Smad4NT under TGF‐β1 stimulation	^143^
	A549 cells	Kaempferol	25 μM	Decreased GST expression, NQO1, HO1, triggered Nrf2 by tBHQ	^146^
	NSCLC cells	Kaempferol	20, 40, 60, 80, 100, 120, 140 μM	Reduced migration, metastasis, induced cell mortality, apoptosis, modulated EMT protein expression	^147^
In vivo	BALB/c mice injected A549 SP cells	CUR	100 mg/kg, every other day	Reduced tumour weight, size, down‐regulated Notch HIF‐1 mRNA, VEGF, NF‐κB expression	^105^

Abbreviations: DNA‐DRP, DNA damage/repair pathway; MAPK, mitogen‐activated protein kinase; MTP, mitochondrial transmembrane potential; NCI‐H292, human lung cancer cell; NF‐κB, nuclear factor kappa B; Ref, references; Smad3PCPC, Smad3 phosphorylation, C‐terminus phosphorylation, complex; Smad4NT, Smad4, nuclear translocation; tBHQ, tert‐butylhydroquinone; VEGF, vascular endothelial growth factor.

##### Extract preparation

*C. longa* rhizome powder (100 g) was dissolved in 200 mL n‐hexane and the solution was shaken for 4 h at 45°C. Then, the supernatant was transferred to a tube. This step was performed three times. Then, the residue of n‐hexane extraction was dissolved in 200 mL dichloromethane instead of n‐hexane and the same steps were repeated. The debris of extraction with dichloromethane was dissolved in 200 mL methanol and in the same way, supernatant was collected. Finally, the solvents of all three phases were dried by rotatory‐evaporator and the remaining powders were stored at—20°C until used.^103^

##### Growth‐inhibitory activity in cell culture

N‐hexane extract of *C. longa* at concentrations of 0.114, 0.142, 0.171 and 0.199 mg/mL inhibited telomerase in A549 lung cancer cells dose‐dependently.^103^ Administration of CUR (1%), in diet before and during weekly NTHi exposure in mice, significantly decreased lung tumours number in the absence of NTHi exposure and in the presence of NTHi exposures (85% and 53% respectively). In addition, direct anti‐tumoral effects of CUR was reported in in vitro of murine K‐ras‐induced lung adenocarcinoma cell lines (LKR‐10 and LKR‐13) through reduction of cell viability, colony formation and apoptosis induction.^104^ In addition, tumour size and weight, Notch and HIF1 mRNA and tumour VEGF and NFB expression were all decreased by CUR (100 mg/kg). VEGF signalling was also inhibited by CUR, consequently inhibiting cancer cell growth.^105^

CUR (0, 5,10, 20 and 40 μM) applied to lung cancer stem cells for 7 days acted as an interventional agent by inhibiting both the Wnt/ß‐catenin and the Sonic Hedgehog pathways.^106^ CUR inhibits Wnt and mTOR pathways by down‐regulating urothelial cancer‐associated 1 (UCA1).^107^ CUR (25–100 μM, for 6–48 h) also inhibited lung cancer cell growth by down‐regulating UCA1, and by enhancing miRNA‐98 activity.^108^

Both pure CUR and nanoencapsulated CUR (CUR‐Fe3O4) (0–120 mM, 24 and 48 h) reduced proliferation and hTERT gene expression in lung cancer cells but CUR‐Fe3O4 was most effective.^109^ Additionally, CUR‐Fe3O4 decreased migration of human lung cancer cells and ROS‐mediated apoptosis.^110^ Lung cancer cells in 160 mM CUR for 12 to 72 h showed increased p53, BCL‐2 (promoter of apoptosis), BCL‐XL (promoter of apoptosis), Bak (promoter of apoptosis) and Cas gene expression.^111^ CUR (10 M) has also been found to regulate axon guidance, glioma, ErbB tyrosine kinase receptor signalling pathways and reduce metastasis in lung cancer cells.^112^

As a result of increasing FOXA2 expression and modulating STAT3 signalling pathways, CUR treatment (40 μ M, 4 h) inhibited human NCI‐H292 cell growth.^113^ In NCI‐H460 cells, CUR causes DNA repair and DNA damage.^114^ CUR (20 and 40 mM) reduced tumorspheres formed by H460 lung cancer cells and also inhibited proliferation and colony formation of the cancer cells. The inhibitory action of CUR on the JAK2/STAT3 pathway is implicated in the reduction of tumour spores and the suppression of tumour growth in mice bearing lung cancer xenografts.^115^

Gefitinib is a medication for cancer that inhibits EGFR in target cells. In primary NSCLC cell lines that are gefitinib‐resistant (H157 and H1294), CUR reinstated the anti‐cancer effects of gefitinib.^116^ CUR also inhibited SCLC cells, up‐regulated the cyclin‐dependent kinase inhibitors: p27 and p21 and down‐regulated cyclin D1.^117^ Additionally, CUR activated the ERK1/2 signalling pathway to increase the expression of forkhead box protein O1 (FOXO1).^117^ PI3K/Akt/mTOR was also inhibited by CUR, consequently inhibiting the growth of NSCLC cells by inducing autophagy and apoptosis^118^, ^119^, ^120^, ^121^ and modulating calcium signalling.^122^ CUR also inhibits adiponectin receptor 1, preventing cancer cells from migrating and invading their host.

In vitro studies have shown that CUR (10 μ M for 4 days) induces the maturation of lung cancer patient‐isolated regulatory cells (Treg) to T helper (Th)‐1 cells by inhibiting DNA transcription of forkhead protein‐3 (Foxp3) and increasing expression of IFN‐γ. Treatment with CUR in lung cancer patients led to a significant increase in peripheral Tregs compared to healthy subjects.^123^ Treatment of A549 cells with CUR (6 M, for 48 h) increased the expression of the enzyme EZH2 through microRNAs (miR)‐7c and miR‐101 and reduced the expression of NOTCH1 via EZH2 activation.^124^ CUR (0, 15, 30, 45 and 60 μ mol/L) significantly inhibited lung cancer invasion and metastasis by inhibiting GLUT1/MT1‐MMP/MMP2 pathways and was also found to significantly inhibit GLUT1/MT1‐MMP/MMP2 expression and invasion in mice.^125^ Wang et al (123) demonstrated that CUR‐induced reduction in NSCLC (A549) viability is related to oxidative stress and its cytotoxicity by causing ROS accumulation.^126^ In the same study, CUR prevented NSCLC proliferation by modulating the Wnt/ß‐catenin pathway (123). Furthermore, by reducing mitochondrial transmembrane potential, CUR (0, 10, 20 and 30 μM, for 2 h) activated the mitochondrial apoptosis and DNA damage repair pathways.^127^ MHMM‐41 (8 μM and 16 μM for 12 h) exerted even better antiproliferative effects on A549 cancer cells and also induced ROS‐mediated apoptosis and migration of A549 cells.^128^ CUR (30 μM) inhibited migration and tube formation in NCI‐H460 cells but this could be reversed by infecting the cells with the dominant‐active variant STAT3C. This result suggests that CUR's inactivation of STAT3 is responsible for its effect on tumour angiogenesis.^129^ LCLC 801D cells were effectively inhibited from migrating and invasively spreading by low‐toxic levels of CUR that disrupted Rac1/PAK1 signalling pathways and MMP‐2 and MMP‐9 expression.^130^

A study was performed using CUR dry powders and liposomal (LCDs) against human lung cancer A549 cells and normal human bronchial epithelial BEAS‐2B cells. CUR and LCDs reduced TNF‐α levels in bronchoalveolar lavage fluid) BALF, (malondialdehyde (MDA) and Cas‐3 levels in lung tissue and expression of VEGF and B‐cell lymphoma 2 (BCL‐2). However, CUR powder showed a much stronger anti‐cancer effect than LCDs.^131^ Treatment with CUR also reduced activation of HLJ1 via activation of the JNK/JunD pathway and also led to increased expression of E‐cadherin in lung cancer cells.^132^

##### Anti‐tumour activity in vivo

In an animal model of lung cancer, the anti‐carcinogenic effect of CUR (100 mg/kg, 0.2 mL, once every other day) was shown by reduced tumour volume and weight, down‐regulated Notch and HIF1 mRNA expression and inhibited VEGF and NFB.^105^ In another study conducted on mice, CUR administration reduced the number of visible lung tumours and inhibited NTH's ability to increase neutrophil chemokine levels.^133^ In a rat model of lung cancer, administration of CUR and resveratrol decreased Cyt c activity and oxidative enzymes, increased lipid peroxidation, SOD and CAT activities and reduced LPO.^134^ CUR liposomes increased apoptosis in cells and adiponectin expression and tumour growth in vivo were both inhibited by CUR treatment. CUR inhibited adiponectin expression by inhibiting NF‐ĸ B/MMP pathways, resulting in decreased migration and invasiveness of the A549 cancer cells.^135^

In both in vitro and in vivo studies, *C. longa* and CUR have been found to suppress cancer cell proliferation and induce apoptosis of lung cancer cells. Additionally, CUR altered the expression of EGFR, microRNAs and autophagy in cancer stem cells. It is believed that CUR influences the expression of different genes in the cancer cells themselves, such as NF‐kB, STAT‐3 and AP‐1, as well as protein kinases, including MAPK and enzymes such as COX and LOX. Thus, CUR makes for a promising therapeutic target in treating lung cancer.

#### 3.1.7 Achillea millefolium

*Achillea millefolium* (*A. millefolium*), also known as yarrow, is a member of the Asteraceae family and found across Europe, Asia, North Africa and North America.^136^, ^137^

The anti‐inflammation and antioxidant and improvement effects of *A. millefoliumi* on pain and estrogenical wounds were reported and chronic administration (ED50 = 32 mg/kg، p.o) increased above the affects in rats for 90 days but it did not show any serious side effect or toxicity.^138^ In review articles, it was stated that *A. millefolium* extracts are safe in the concentrations used in cosmetics.^139^ Several in vitro studies have explored the anti‐tumour potential of *A. millefolium* and its constituents, and the findings of *A. millefolium* effects on lung cancer are summarized in Table 3.

##### Extract preparation

The lyophilized powder (1 g) of the plant was extracted with 40 mL of methanol by stirring (25°C, at 10,000 x g) for 1 h and filtered through filter paper. The extract was evaporated at 40°C in rotary to dryness. The ethanolic extract was prepared using the dry fruiting bodies (1 g) with 30 mL of 90% ethanol by stirring for 48 h at 70°C. The extract was filtered and centrifuged to get a clear liquid and evaporated at 40°C.^140^

##### Growth‐inhibitory activity in cell culture

Administration of two doses of *A. millefolium* extract on human lung cancer cells (NCI‐H292) increased the level of apoptosis, up‐regulated p53 protein and Bax, activated Cas‐3 protein expression and increased activity of the ER stress pathway, GRP78, Cas‐12 and Cas‐8.^141^ Similarly, *A. millefolium* extract (75 and 100 μg/mL) altered the cycle profiles, increased apoptosis and increased p53 and p21 expression in HCT‐15 and NCI‐H460 cells.^140^ Administration of kaempferol (17.5, 35, 52.5, 70 μM) inhibited Akt‐1 phosphorylation and MEK1/2, activated mitogen‐activated protein kinase (MAPK), induced cell apoptosis via cleavage of Cas‐7 and induced poly ADP‐ribose polymerase (PARP) activity in A549 cells.^142^ In addition, kaempferol (10, 25, 50 μM) treatment of A549 lung cancer cells, followed by TGFß‐1 stimulation, resulted in phosphorylation of Smad3 (although phosphorylation at residue Thr179 was down‐regulated) and the formation and nuclear translocation of Smad4 complexes.^143^ Kaempferol treatment combined with radiation therapy to kill tumours increased the amount of tumour death, both in vitro and in vivo, by suppressing the AKT/PI3K and ERK pathways and activating the mitochondria apoptosis pathway.^144^ In the same study, kaempferol was also found to induce G2/M cell cycle arrest, inhibit clonogenic survival and elevate tumour cell apoptosis. Administration of kaempferol‐3‐O‐rutinoside significantly stimulated cancer cell cytoskeleton collapse, mitochondrial dysfunction and apoptosis via the calcium signalling pathway.^145^ Kaempferol treatment (25 μM) for 48 h also decreased the expression of GST, NQO1 and HO1, triggered Nrf2 by tert‐butylhydroquinone (tBHQ), reduced ROS levels and induced apoptosis in treated cells.^146^ Finally, Hang et al^147^ found various doses of kaempferol (20, 40, 60, 80, 100, 120 and 140 μM) reduced migration and metastasis of cells, induced cell mortality and apoptosis and modulated EMT protein expression in human NSCLC cells. These results suggest that the anti‐cancer mechanisms of *A. millefolium* and its constituent kaempferol are broad and lead to cancer cell immobility and death. Compared to the other plant species discussed, *A. millefolium* has also been reported to disrupt the cytoskeleton of lung cancer cells.

#### 3.1.8 *Portulaca oleracea*e

*Portulaca oleracea (P. oleracea)* belongs to the Portulacaceae family and grows in Iran, India, Japan, China and southern Europe.^148^ Genistein and luteolin^149^ are natural flavonoid constituents of *P. oleracea* with potential anti‐cancer, antioxidant and anti‐inflammatory properties.^150^ Previous studies showed that *P. oleracea* and genistein treatment reduced inflammation in lung tissue, reduced oxidative stress and improved the Th1/Th2 balance, among other immunological indices.^151^, ^152^, ^153^

Results were reported that administration *P. oleracea* did not show cytotoxic effects in in vitro studies and the IC_50_ value of more than 10 μg/mL but moderate toxic effects were shown in LD_50_ value of 1853 mg/kg.^154^ It was also reported that human consumption of *P. oleracea* poses minimal dangers in humans.^155^ Several studies have reported a therapeutic effect of *P. oleracea*, genistein and luteolin on lung cancer cells, which are discussed below and summarized in Table 4.

**TABLE 4:** The effects of *Portulaca oleracea*, *Allium cepa*, *Brassica juncea* and *Aloe Vera* their constituents, on lung cancer.

Study type	Typ of lung cancer	Preparations	Dose	Effects	Ref.
In vitro	NCIH‐522 cell line	*Portulaca oleracea* whole plant	10–1000 g/μg for 24 h	Reduction in cell viability and changes in cell morphology were recorded	^156^
	NCI‐H209	Quercetin	5 μM	Decreased cell viability, MMP, Cyt c, increased cell cycle, G2/M and subG0/G1 phase cells, cyclin B, Cdc25c‐ser‐216‐p expressions, G2/M arrest, up‐regulated Bax, down‐regulated caspase‐3, poly(ADP‐ribose) polymerase activations cleavage	^179^
	A549 cell	Quercetin	14.5, 29, 43.5, 58 μM	Reduced cell viability, DNA synthesis and Bcl‐2, increased Bax, Bad and Bcl‐x(L)	^180^
	A549 cell line	Genistein	25, 50, 75 and 100 μM	Inhibited cell proliferation, ERK1/2 phosphorylation, PI3K/Akt pathways, down‐regulated MMP‐2 expression	^159^
	H460 non‐small‐cell	Genistein	5, 10, 20, 30 and 50 μM	Increased Bax expression, induction of p21 protein, induced apoptosis via the p53‐independent pathway	^160^
	A549 human lung cancer	Genistein	5–200 μM	Reduced miR‐27a, MET protein expression	^161^
	A549 cells	Sinapic acid	5–10,000 μg/mL	Decreased necroptotic marker proteins cyclophilin A HMGB1	^195^
	H460 cells	Aloe‐emodin	20 μM	Increased apoptosis or necrosis, cell death, MAP kinase members activation MPTP, changed Cyt c protein expression	^200^
	A549 and NCI–H1299 cells	Aloe‐emodin	10, 20, 40 μM	Stimulated autophagy through activation of MAPK, inhibited Akt/mTOR pathway, reduced ROS, mediated autophagy	^201^
	CH27	Aloe‐emodin	40 μM	Increased Cyt c, Bcl‐2 family protein expression such as Bcl‐XL, Bag‐1 and Bak	^203^
	H460 cell	Aloe‐emodin	20 μM, 2 h	Increased PKCδ expression, cell death, decreased CRP expression, including sarcoma RAS, RHO, p38, HSP27, FAK, α‐actinin, tubulin	^204^
	H460 cell	Aloe‐emodin	50 μM, 16 h	Increased cAMP‐dependent protein kinase, protein kinase C, Bcl‐2, caspase‐3, p38	^206^
In vivo	Nicotine treated rats	*A. cepa oil*	100 mg/kg, 21 days	Increased CAT and SOD activity	^182^
	B(a)P‐induced LC in mice	Sinapic acid	30 mg/kg, orally	Declined IgG, IgM levels, leukocyte counts, lipid peroxidation, pro‐inflammatory cytokines, AHH, LDH, GGT, 5′NT, CEA	^194^

Abbreviations: A549 cell line, human non‐small cell lung cancer cells; B(a)P, benzo(a)pyrene CAT, catalase; Bax, B‐cell lymphoma 2‐associated X protein; Bcl‐2, B‐cell lymphoma 2; CRP, cytoskeleton‐related protein; FAK, focal adhesion kinase; HMGB1, high mobility group box 1; HSP27, heat shock protein 27; LC, lung cancer; miR‐27a, microRNA‐27a; MMP, mitochondrial membrane potential; MPTP, mitochondrial permeability transition pore; PKCδ, protein kinase Cδ; Ref, references; RHO, ras homologue gene family member.

##### Extract preparation

The oil from dried and grounded *P. oleracea* seeds was extracted by continuous extraction in Soxhlet apparatus for 12 h using petroleum ether (60–80°C boiling range) as a solvent. Then the solvent was evaporated and kept at −4°C.^156^, ^157^

##### Growth‐inhibitory activity in cell culture

Following treatment of HepG2 and A‐549 cells with *P. oleracea* treatment (10–1000 ng/mL m for 24 h), a dose‐dependent reduction in cell viability and changes in cell morphology were recorded. In addition, the lung adenocarcinoma cell line (A549) showed decreased proliferation and a higher apoptotic rate compared to controls.^156^

Considering mRNA and protein levels, glutein therapy increased Bax but decreased Bcl‐2 (anti‐apoptotic factor) in A549 cells.^158^ Similarly, genistein may enhance the production of Bax and down‐regulate Bcl‐2, as well as inhibit lung cancer cell proliferation and induce their apoptosis.

The treatment resulted in inhibited tumour growth by activating miR‐128 in the tumour cells. *P. oleracea* cytotoxic effects on human lung (K562 and A549) and breast cancer (MCF‐7 and MDA‐MB‐435) cells were examined using four alkaloids isolated from air‐dried aerial parts. The alkaloids isolated from *P. oleracea* showed moderate cytotoxic activity against A549 cells and only weak cytotoxic activity against K562 cells.^157^ The alkaloids did not appear to have any cytotoxic effects on MCF‐7 and MDA‐MB‐435 (breast cancer) cells.

Genestein (25, 50, 75 and 100 μM) also suppressed cell proliferation and reduced MMP‐2 expression in human NSCLC cells (A549 cell line) in a dose‐dependent manner.^159^ As a result, ERK1/2 and PI3K/Akt phosphorylation was inhibited, leading to these protective effects against lung cancer. Furthermore, genistein (5, 10, 20, 30 and 50 μM) increased Bax expression and p21 protein levels in H460 NSCLC and H322 cells, independent of the p53 pathway.^160^ Moreover, genistein (5, 10, 25, 50, 100 and 200 μM) exerted anti‐cancer effects on the A549 human lung cancer cell by regulating Cas‐3/9 activity, miRNA27a activity and the expression of the MET protein.^161^ A549 cells, expressing TRAIL‐resistant human adenocarcinoma genes, were treated with genistein (0, 10, 20 and 40 μM) which led to enhancing p62, activating Cas‐3 and Cas‐8 and increasing TRAIL‐induced tumour cell death by activated autophagy.^162^ Apoptosis and autophagy of NSCLC cells were also increased by genistein in vitro by inhibiting Bcl‐xL distribution in the cytoplasm, lowering cytoplasmic Bcl‐xL levels and increasing Bcl‐xL and Beclin‐1 dissociation.^163^ Bcl‐xL distribution and levels in the cytoplasm and Bcl‐xL and Beclin‐1 dissociation are closely associated with NSCLC radiosensitivity by facilitating DNA damage‐induced apoptosis and autophagy. Therefore, genestein could be considered as a therapeutic agent for enhancing the sensitivity of radiation therapy in NSCLC patients.

#### 3.1.9 Allium cepa

*Allium cepa L*. (*A. cepa*), or onion, belongs to the Liliaceae family and includes over 250 genera and 3700 species.^164^, ^165^, ^166^ Also, Garlic (*Allium sativum*) is a member of the onion (Amaryllidaceae) family and is classified in the same genus to which onion, leek, chive and shallot belong.^167^ The plant grows all over the world but is most common in zones with moderate climates and originates from central Asia.^168^, ^169^ Anti‐fungal,^170^ anti‐cancer, anti‐inflammatory,^171^ antioxidant, antispasmodic,^168^, ^172^ antimicrobial, anti‐mutagenic,^173^ antidiabetic,^174^, ^175^ antiplatelet^176^ and anti‐asthmatic properties^177^ of its main constituent quercetin (QT) have been reported.

To our knowledge, there is no published data regarding the safety of *A. cepa* in humans. Several studies have found *A. cepa* and CUR works against lung cancer cells and the potential mechanisms are explored below and summarized in Table 4.

##### Extract preparation

Onion bulbs (100 g) was peeled, washed, cut into pieces and crushed and percolated in 500 mL of distilled water for 3 days with intermittent shaking. Using Whattman no.1 filter paper the extract was filtered to get fine.^178^

##### Growth‐inhibitory activity in cell culture

NCI‐H209 lung cancer cells treated with QT (5 μM) glucuronides are less likely to survive but they exhibit a higher percentage of cells in the G2/M phase, subG0/G1 phase and in the G2/M phase over time.^179^ Raising cyclin B expression and glucuronic acid, Cdc25c‐ser‐216‐pand Wee1 levels were also reduced, indicating G2/M arrest. Several features of apoptosis were also observed after QT treatment, including the release of Cyt c, the induction of Bax and Bcl‐2, the activation of Cas‐3 and the cleavage of poly (ADP‐ ribose) polymerase.^179^ Increases in Bax, Bad and Bcl‐x (L) levels occurred dose‐dependently following treatment with QT (14.5, 29, 43.5, 58 μM).^180^ In the same study, QT was also found to inhibit Akt‐1 and p‐Akt‐1, phosphorylated ERK and MEK1/2 in a dose‐dependent manner. Several studies have indicated that QT is capable of causing apoptosis via activation of caspase‐3 in A549 lung carcinoma cells.^180^ Researchers found that consumption of QT‐rich foods negatively correlated with lung cancer risk in non‐tumour lung tissue from 38 adenocarcinoma patients. This correlation was not different between genotypes of P450s or GSTs, gender or subtypes of lung cancer, and the correlation was strongest in smoker subjects (who smoked >20 cigarettes per day).^179^ Furthermore, high consumption of QT‐rich foods led to higher expression of GSTM1, GSTM2, GSTT2 and GSTP1 relative to the low consumption group, but there was a lower expression of some P450 genes. According to these findings, QT intake is able to reduce smoking‐induced lung cancer risk.^179^

A QT‐containing diet is also associated with poorer miRNA expression profiles of the tumour suppressor let‐7 family in lung cancer tissue samples from 264 cases (144 adenocarcinomas and 120 squamous cell carcinomas).^181^ QT‐rich diet also significantly differentiates miR‐146, miR‐26 and miR‐17, which are cancer‐related miRNAs. It was also found that 33 miRs were differentially expressed between high and low QT‐rich food consumers in former and current smokers with adenocarcinoma. QT‐rich foods in adenocarcinoma demonstrate differential expression of biologically functional miRs in this study, indicating that QT may have a therapeutic effect on lung cancer. Crocin (1, 2, 4, 8 and 16 mg/mL for 24 and 48 h) reduced mRNA expressions of B‐cell lymphoma 2 and Bax, but increased expressions of p53. Two lung cancer cell lines showed that QT also inhibited proliferation.^71^

##### Anti‐tumour activity in vivo

*A. cepa* extract (10 g/L) showed an antiproliferative effect and mitotic indices appeared to be reduced in association with concentrations of the extracts.^178^ Moreover, the extract showed no anti‐mutagenic or genotoxic properties. It is possible that the effects of onion extracts are caused by phenolic compounds. An antioxidant effect of *A. cepa* oil (10 mg/kg for 21 days) was shown in rats that had been exposed to nicotine, with higher levels of catalase (CAT) and superoxide dismutase (SOD) noted.^182^ Administration of crocetin (50 mg/kg for 8 and q8 weeks) reduced cell proliferation, glycoprotein and polyamine synthesis in B(a)P‐induced lung cancer in mice.^183^

The anti‐cancer effects of *A. cepa* and its main constituent QT reported in the above studies indicate that *A. cepa* and QT could inhibit adenocarcinomas parameters by modulating the expression of B‐cell lymphoma, Bax mRNA, Bcl‐2 mRNA, cell viability and DNA synthesis in lung cancer cells.

#### 3.1.10 Brassica juncea

*Brassica juncea* (*B. juncea*), or mustard leaves, is a cruciferous vegetable consumed in different Asian and African countries.^184^, ^185^, ^186^ Different biological activities of mustard and its constituents, such as antioxidant,^185^, ^187^, ^188^ anti‐inflammatory^189^ and anti‐depressant activities^190^ have been reported. *B. juncea* has also demonstrated cytotoxic effects against cancer cells from different organs, including the colon, stomach, lungs and breast.^191^

Adminstration of *F. suspensa* extract did not show hepatotoxicity, hemolytic activity or other side effects indicating its safety when used at the studies doses.^192^ The effects of mustard and its constituents on lung cancer are summarized in Table 4.

##### Extract preparation

Dried *B. juncea* powder (20 g) was dissolved in 100 mL of 100% methanol and kept in a rectangular double‐walled water bath at 50°C for 4 h. Then the excerpt was filtered with filter paper (diameter of 125 mm) and the filtrate was kept at 50°C until it turned into a semisolid form.^193^

##### Growth‐inhibitory activity in cell culture

DPPH assay analysis found that a combination of *A. deliciosa* and *B. juncea* extracts increased antioxidant and antiproliferation activity markers and apoptosis in human lung carcinoma cells.^193^ Treatment of lung cancer cells with sinapic acid decreased potential cytotoxic and apoptosis activity, reduced ROS production levels and elevated Cas activity (Cas‐3 and Cas‐9).^194^ Similarly, administration of sinapic acid (500, 1000, 2000, 5000 and 10,000 μg/mL) to A549 cells was found to increase Cas‐3 activity, as well as lactate dehydrogenase (LDH) activity and decrease necroptotic marker proteins, cyclophilin A and high mobility group box 1 (HMGB1).^195^

##### Anti‐tumour activity in vivo

In vivo results showed that treatment with sinapic acid (30 mg/kg in corn oil, orally) improved lung and body weight, reduced levels of IgG, IgM and leukocyte counts, reduced the function of neutrophils, lipid peroxidation, pro‐inflammatory cytokines, AHH, LDH, GGT, 5′NT and CEA and elevated the phagocytic index, activity index and activity of antioxidant enzymes.^194^

The above in vitro and in vivo results indicate that mustard and its constituents could be effective in lung cancer treatment via increasing antioxidant activities and antiproliferation activity markers, apoptosis and improving levels of immunoglobulins, Cas, HMGB1, LDH and pro‐inflammatory cytokines in lung cancer models.

#### 3.1.11 Aloe vera

*Aloe Vera* (*A. vera*) is used to treat wounds in traditional Chinese medicine but is also widely used as a food, a remedy for constipation, wound healing and anti‐cancer purposes in Egypt, Greece and China. These medicinal properties have also been tested in pharmacological studies.^196^, ^197^, ^198^

Regarding the safety of the plant, administration of tree doses (832.5, 1665 and 3330 mg/kg/bw by gavage), did not show any toxicity and mortality.^199^ Several studies have indicated an effect of *A. vera* and its derivatives on lung cancer and the findings are discussed below and summarized in Table 4.

##### Growth‐inhibitory activity in cell culture

Administration of *Aloe emodin* (*A. emodin*, 20 μM) to lung cancer (H460) cells increased apoptosis, necrosis and cell death, increased MAP kinase membrane activation of a mitochondrial permeability transition pore, changed Cyt c protein expression and mediated localization of F‐actin.^200^ In addition, treatment of A549 and NCI–H1299 cells with *A. emodin* (10, 20 and 40 μM) stimulated autophagy through activation of MAPK signalling, inhibition of the Akt/mTOR pathways, reduced ROS levels and mediated autophagy.^201^ A study looking at the impact of *A. emodin* on CH27 and H460 cells found apoptosis was induced via nuclear morphological changes and DNA fragmentation, cystolic Cyt c fractions were enhanced, Cas‐3 was activated and protein kinase C (PKC) expression was increased.^202^ Similarly, Lee et al^203^ found *A. emodin* (40 μM) treatment of CH27 cells also triggered apoptosis by DNA fragmentation and enhanced expression of Bcl‐2 family proteins such as Bcl‐XL, Bag‐1 and Bak which increased Cyt c in the cytosolic fraction. *A. emodin* administration (20 μM for 2 h) to H460 cells also stimulated protein kinase Cδ (PKCδ) expression and cell death and decreased expression of cytoskeleton‐related proteins, including sarcoma (RAS), RAS homologue gene family member A (RHO), p38, heat shock protein 27 (HSP27), focal adhesion kinase (FAK), α‐actinin and tubulin.^204^ Lee et al^205^ found the treatment of carcinoma H460 cells with *A. emodin* (40 μM) increased the amount of proform and fragments of nucleophosmin, inducing apoptosis. In a more recent study, apoptosis‐inducing proteins such as cAMP‐dependent protein kinase, protein kinase C, Bcl‐2, Cas‐3 and p38 were also measured in carcinoma H460 cells following treatment with *A. emodin* nanoparticles (50 μM) for 16 h.^206^ These *A. emodin* nanoparticles cleaved Cas‐3, Poly (ADP‐ribose) polymerase (PARP), Cas‐8 and Cas‐9 and simultaneously activated MAPKs and inhibited PI3K/AKT, leading to marked inhibition of cancer cell proliferation, induced cell cycle arrest and apoptosis.^206^

Based on the above studies, *A. vera* and *A. emodin* showed anti‐carcinoma effects on lung cancer cells. Therefore, *A. vera* and *A. emodin* could be effective in lung cancer therapy by inhibiting cell proliferation, cytoskeleton activation, stimulated cell apoptosis as well as Cas‐3 and PKC activation, MAPK signalling, inhibition of ROS production, Akt/mTOR and PI3K/AKT pathways.^206^

### 3.2 Different mechanisms of natural products against lung cancer

In the present article, the effects of the most effective 11 medicinal plants and their constituents against lung cancer were reviewed. The anti‐cancer effects of the above medicinal plants and their derivatives on lung cancer were mediated through different mechanisms of action including inhibition or abnormal cell growth and proliferation, cancer cell apoptosis and death, oxidative stress and inhibition of migration and or metastasis.^37^, ^207^, ^208^, ^209^, ^210^ Below we summarize the mode of action and molecular strategies of medicinal plants and their derivatives against lung cancer, as evidenced by empirical study.

#### 3.2.1 Cell proliferation, cell viability and tumour growth

**FIGURE 2:**
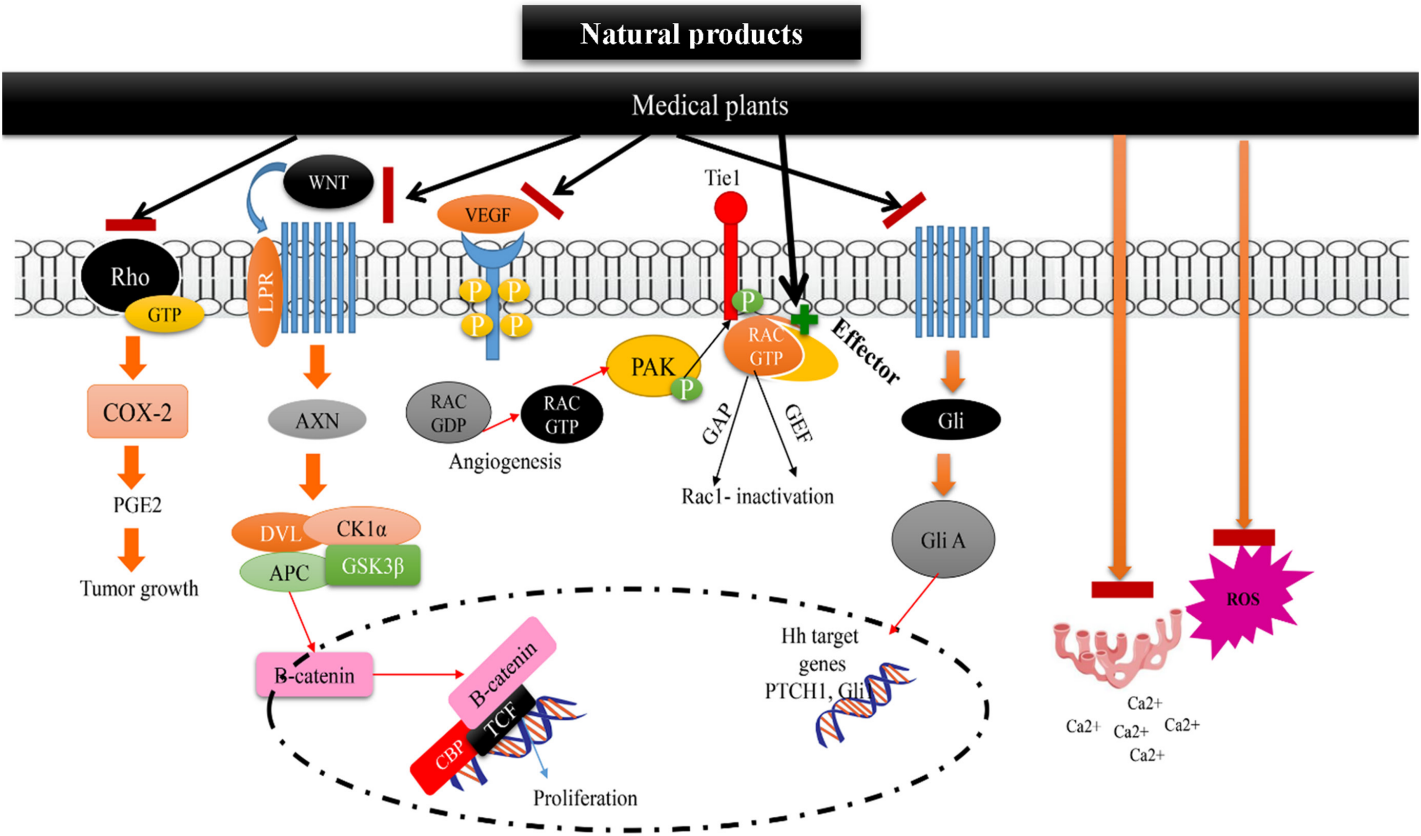
Anti‐apoptosis, cell death and anti‐cell migration mechanisms of natural products on lung cancer.

In lung cancer, increasing cell proliferation and viability of cancer cells demonstrates the advancement of the disease. Therefore, the effect of natural products on lung cancer cell proliferation and viability is an indicator of their therapeutic effects. The in vitro and in vivo studies demonstrated the inhibitory effects of *A. vera*, *C. longa*, *C. sativus*, *B. juncea*, *A. cepa*, *P. oleracea*, *Z. multiflora*, *N. sativa* and their constituents on lung cancer cell proliferation.^43^, ^45^, ^68^, ^81^, ^115^, ^127^, ^156^, ^159^, ^180^ In addition to cell proliferation and viability, tumour growth rate is used to diagnose the stage and severity of cancer. *A. vera*, *C.longa*, *F. szowitsiana*, *P. oleracea* and *Z. multiflora* all demonstrated an ability to block or disrupt tumour growth in lung cancer models and cell lines.^39^, ^90^, ^131^, ^135^, ^211^ Figure 2 illustrates the effects of these natural products on cell proliferation and tumour growth in lung cancer.

#### 3.2.2 Apoptosis

Increased apoptosis reduced rates of lung cancer cell proliferation, growth and prevented metastasis, making it a powerful effect against lung cancer. Several studies reported that the natural products: *O. basilicum*, *P. oleracea*, *A. vera*, *C. longa*, *A. cepa*, *C. sativus*, *N. sativa*, *Z. multiflora* and their constituents increased the level of apoptosis in lung cancer models or cell lines.^58^, ^86^, ^128^, ^142^, ^146^, ^161^, ^180^, ^206^ How these natural products induce apoptosis in lung cancer is shown in Figure 2.

#### 3.2.3 Growth of lung cancer

The extracellular matrix (ECM) is a crucial component of the tumour microenvironment (TME) and is present in both interstitial and epithelial vessels. It plays a dual role in cancer progression, as it facilitates interactions between cancer cells and stromal cells, promoting carcinogenesis, while also acting as a barrier against tumour metastasis.^212^ Degradation of the ECM allows cancer cells to traverse it and enter blood vessels. Subsequently, with the assistance of certain cytokines, cancer cells can pass through vessel walls and extravasate to secondary sites, where they continue to proliferate and form metastatic lesions.^213^ Matrix metalloproteinases (MMPs), a family of zinc‐dependent endopeptidases produced by various cell types, including fibroblasts, epithelial cells and immune cells, are the primary proteases responsible for ECM degradation. The urokinase‐type plasminogen activator (u‐PA) is a key enzyme involved in ECM degradation and the activation of proMMPs, including MMP‐2. Abnormal expression of membrane type 1‐matrix metalloproteinase (MT1‐MMP) in tumours is associated with the regulation of MMP‐2 activity.^214^ MMP‐9, another member of the MMP family, has been identified as a biomarker for various cancers.^215^ Therefore, inhibiting the activity or expression of MMPs may be a potential strategy to suppress tumour invasion and metastasis. In lung cancer and other cancers, overexpression of GLUT1 (glucose transporter 1) is associated with poor prognosis. CUR, a natural compound with diverse activities, has been studied for its potential therapeutic effects.^216^ Liao et al. demonstrated that CUR treatment reduced the expression of GLUT1, MT1‐MMP and MMP2 in A549 lung cancer cells. Conversely, in GLUT1‐overexpressing A549 cells, CUR's anti‐migration and anti‐invasion effects were impaired, and MT1‐MMP and MMP2 expression were up‐regulated. Consistent with these findings, in vivo experiments using nude mice showed that CUR treatment significantly reduced metastatic rates in A549 cells, but this effect was hindered in GLUT1‐overexpressing cells. The study concluded that CUR suppresses migration and invasion by modulating the GLUT1/MT1‐MMP/MMP2 pathway in A549 cells.^125^ Another compound, honokiol, derived from *Magnolia officinalis*, was found to inhibit migration and invasion in H1299 lung cancer cells by disrupting the expression of MMP‐9 and the Hsp90/MMP‐9 interactions mediated by HDAC6. Honokiol promoted the degradation of MMP‐9 through the ubiquitin‐proteasome pathway, rather than inhibiting its transcription. HDAC6, a deacetylase, regulates the stability of Hsp90, which is involved in the activation of MMP‐2/9. Honokiol inhibited the expression of acetyl‐α‐tubulin, a specific substrate of HDAC6, and further experiments confirmed the regulation of MMP‐9 by HDAC6.^217^ Other natural compounds, such as theaflavin and theaflavin digallate from black tea, were found to exert anti‐metastasis effects by inhibiting type IV collagenase in mouse LLC cells.^218^ (‐)‐Epigallocatechin‐3‐gallate (EGCG), a polyphenol found in green tea, has also been extensively studied. Deng et al. reported that EGCG inhibits invasion in CL1‐5 lung cancer cells by suppressing the mRNA and protein levels of MMP‐2 through the JNK pathway. EGCG was also found to enhance the anti‐cancer effects of docetaxel and reduce MMP‐2 expression. Additionally, EGCG was shown to suppress migration and invasion by inhibiting epithelial‐mesenchymal transition (EMT) and angiogenesis induced by nicotine.^219^, ^220^, ^221^ Another important target of cancer treatments is decreasing the amount of tumour cell migration and metastasis. There are several in vitro and even in vivo studies that found *A. millefolium*, *C. longa*, *Ferula* spp., *N. sativa* and their constituents reduced lung cancer cell migration^44^, ^58^, ^81^, ^91^, ^130^, ^143^ and lung cancer metastasis.^58^, ^81^, ^112^, ^125^, ^147^ The effects of these natural products on migration and metastasis of lung cancer are shown in Figure 2.

#### 3.2.4 Signalling pathways underlying the growth‐suppressive activity of medicinal plants and their constituents

Studies investigating various types of cancer have revealed some of the molecular events behind cancer cell proliferation, viability, migration and metastasis. For example, enhanced tumour growth correlated with increased AMPK and fatty acid oxidation in ovarian cancer cells, linked to dysregulation of mTORC1 expression in renal cell carcinoma and has been connected to the hypoxia and oncogenic HRAS or KRAS pathways in glioblastoma and bladder cancer.^222^

Hence, p53‐deficient KRasG12D NSCLC had dysfunctional mitochondria, lipid accumulation and defects in fatty acid oxidation, resulting in reduced tumour growth.^223^ In addition, mutations in MYC, TP53, Ros‐related oncogenes, the AMPK and PI3K signalling pathways of the NSCLCs enhanced expression of GLUT1, negatively impacting cell proliferation and growth.^223^

In microglial, bladder cancer and HepG2 cells, cell proliferation, migration and survival could be enhanced by up‐regulation of the Gadd45 family member Gadd45β/Myd118, which associates with MKK7/JNKK2, p38 expression, transforming growth factor‐β and TGF‐β via the JAK/STAT pathway, ultimately activating toll‐like receptors and interferon‐γin.^224^, ^225^, ^226^

Increasing accumulation and expression of angiogenesis factors such as VPF and VGEF enhanced metastasis and cell growth in tumour cells. Apoxemia and realizing ROS also triggered angiogenesis factors in human glioma cells.^227^, ^228^ Breast cancer cells tend to proliferate when ß‐actin, FOXM1, FBXW7, Fascin, eNOS, MMP‐2 and HER2‐receptor are expressed or stimulated.^229^ Expressions of Wnt1, β‐catenin and cyclin D1 at the protein level in NSCLC cells increased the risk of mutagenesis, increased cancer cell viability and decreased apoptosis.^230^ Activation of Signal Transducer And Activator Of Transcription 3 (STAT3) signalling induced EMT and increased miR‐193a‐3p, miR‐210‐3p and miR‐5100 levels, prompting lung cancer metastasis.^231^ It was reported that proliferation was suppressed via inhibiting the PHLPP1/AKT pathway.^232^

**FIGURE 3:**
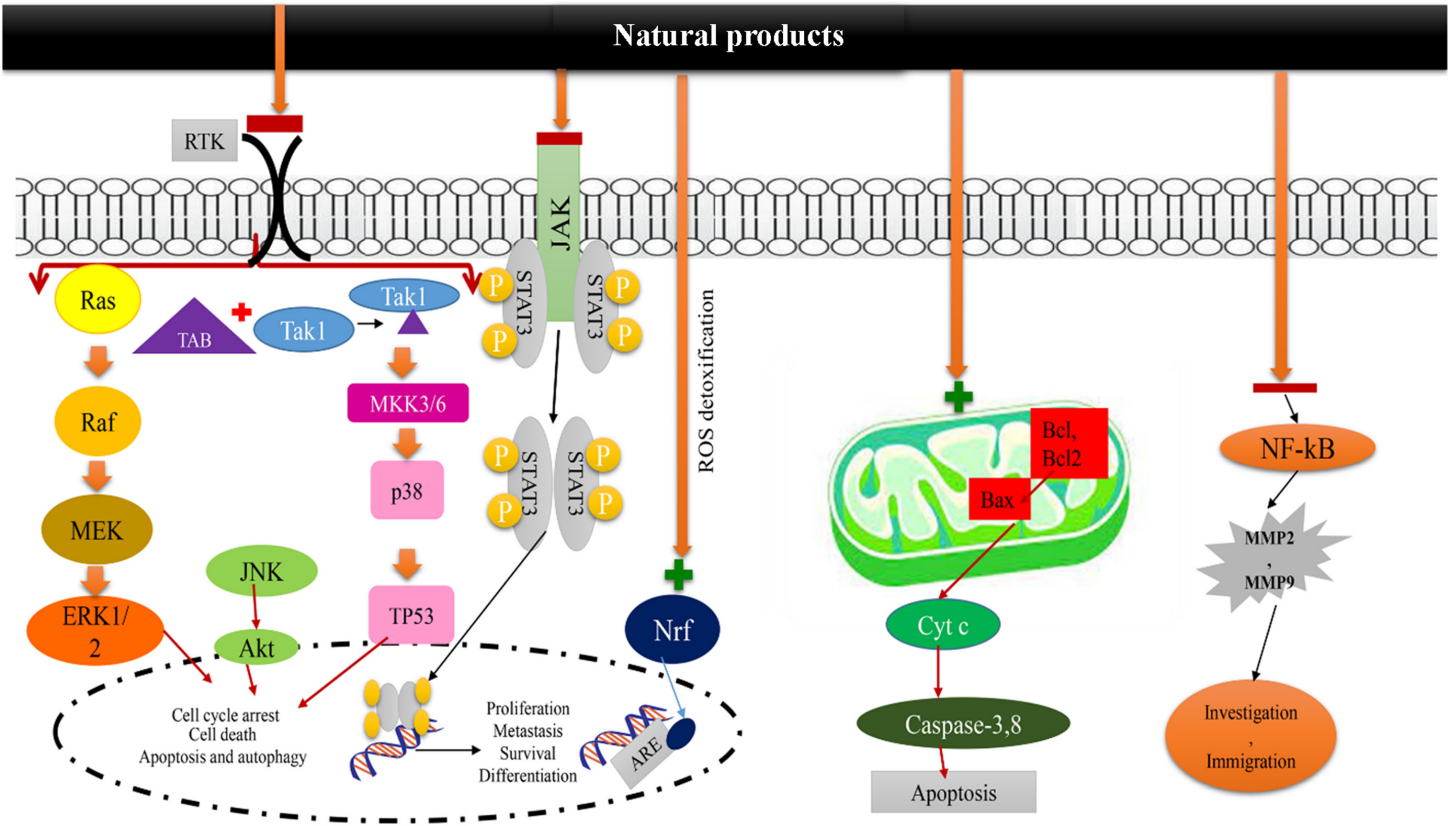
Anti‐cell proliferation, anti‐oxidative and anti‐tumour growth mechanisms of natural products on lung cancer.

The cancer suppression effects of *N. sativa*, *A. emodin*, *F. Szowitsiana and A. millefolium* extracts as well as some derivatives of medicinal plants such as CUR, TQ, carvacrol, genistein, crocin work by increasing apoptosis and authophagy, reducing cell viability or proliferation via RTK and reducing cytotoxicity via inhibition of ROS release and expression.^60^, ^116^, ^204^ The suppression effects of the above plants on P38,^39^, ^116^, ^206^ P53,^37^, ^71^, ^233^ Akt^58^, ^62^, ^159^ and activation of mitochondrial protein expression such as Bcl, Bcl2,^43^, ^56^, ^58^ Cyto C,^43^, ^104^ Cas‐3 and ‐8 down‐regulation of Ca2+ release mediated NF‐κB/MMP^43^ and inhibiting the PI3K/Akt/mTOR, JAK2/STAT3 pathways^135^, ^206^, ^231^ were also shown. *C. longa*, *A. millefolium*, *C. sativus*, *szowitsiana* and CURwere also found to Block the Wnt/β‐catenin and Sonic Hedgehog pathways, reduce metastasis and migration of lung cancer cells via inhibition of NF‐κB and AP‐1, MMp2, JAK/STAT3 pathways, increase FOXA2 expression via regulation of STAT3 signalling pathways, enhance GCL expression via activation of Nrf2, LC3‐II, p62 expression rand decrease cancer cell cytotoxicity via inhibition of ROS and inflammatory markers.^101^, ^113^, ^130^ Therefore natural plant products, including medicinal plants and their derivatives, are promising agents for the treatment of lung cancer by impacting apoptosis, autophagy, cell viability or proliferation, metastasis and migration. The molecular pathways of these anti‐cancer effects are summarized in Figure 3.

#### 3.2.5 Chemosensitization activity

Despite the availability of numerous conventional anti‐cancer drugs with diverse mechanisms of action, the majority of them ultimately induce cell death through necrosis, apoptosis or autophagy by stimulating genotoxic stress.^234^ However, these chemotherapeutic agents primarily target rapidly dividing cells, which often results in adverse effects on normal dividing cells, leading to a multitude of side effects.^235^ While some of these side effects are short‐term, such as nausea, myelosuppression or alopecia, others, including infertility, weight gain, cardiac dysfunction or secondary leukaemia, are long‐term and serious. Additionally, the development of chemoresistance, particularly multidrug resistance (MDR), is another significant obstacle to the success of chemotherapy.^235^ To overcome this challenge, researchers have conducted extensive research on molecules that may enhance the therapeutic index of anti‐tumoral drugs by making tumour cells more sensitive to chemotherapeutic agents, known as chemosensitizers. However, many chemosensitizers have shown toxicity or low efficacy in clinical trials.^236^, ^237^, ^238^

Given these limitations, natural compounds have emerged as a promising source of new molecules for chemosensitization due to their unique structures and mechanisms of action. Many plant‐derived molecules have been found to target multiple pathways involved in cancer and are consumed as food or used as traditional remedies. Natural products have played a significant role in the development of small molecules approved for cancer treatment. For instance, between the 1940s and the end of 2014, 75% of the small molecules approved for cancer treatment were other than synthetic, with 49% being natural products or directly derived from it. Therefore, scientists are currently exploring new molecules, such as natural compounds, to address the important problem of MDR in cancer.^239^, ^240^, ^241^

Natural compounds have emerged as a promising source of new molecules for chemosensitization in cancer therapy due to their unique structures and mechanisms of action. They can be used as chemotherapeutic agents, chemopreventive agents and chemosensitizing agents to improve the effectiveness of conventional chemotherapy.^242^, ^243^ Natural products, especially edible phytochemicals‐nutraceuticals, are attractive partners to be used in combination with chemotherapy due to their high biodiversity, good oral bioavailability and relatively low intrinsic toxicity.^244^ When associated with conventional chemotherapeutics, plant‐based chemosensitizers enhance the cytotoxic effect of the anti‐cancer drugs, promoting a synergistic effect even in cells with acquired resistance. Nutraceuticals represent a specific segment of natural compounds that are consumed as part of a normal diet and are considered pharmacologically safe. However, challenges regarding the limited bioavailability of these compounds have been discussed, and novel formulations and analogues have been developed to increase their efficacy.^52^, ^245^, ^246^ Over the past few decades, numerous studies have indicated that cancer cells can develop resistance to drugs, which can be overcome by combining multiple drugs that act on redundant signalling nodes. EGFRi can initiate various side effects, which can be alleviated by incorporating natural products into the therapeutic regimen.^247^ For instance, Honeysuckle, a natural product obtained from Lonicera japonica Thunb, was shown to reduce acneiform rash incidences and severities induced by EGFRi in lung cancer patients. Additionally, natural bioactive components can help reverse EGFRi resistance in cancer cells. Bruceine H, a derivative of Brucea javanica (L.), was found to overcome resistance to receptor tyrosine kinase (RTK)‐EGFRi in non‐small cell lung cancer (NSCLC) models by suppressing Notch3, EGFR activation and β‐catenin expression., thereby increasing gefitinib response. This combination also induced Foxo3a expression, which correlates with better response to EGFR inhibitors and overall survival in NSLCC patients. Similarly, cryptotanshinone (CTS), a bioactive component of Salvia miltiorrhiza, was shown to sensitize gefitinib‐resistant EGFR‐mutant lung cancer cells by targeting catalase (CAT), heme oxygenase 1 (HMOX1) and stearoyl‐CoA desaturase (SCD). These proteins were identified through proteomic analysis and suggested to be potential therapeutic targets in lung cancers.^248^

## 4 CONCLUSION

This review article presented the potential therapeutic effects of medicinal plants and their constituents on lung cancer based on in vivo and in vitro findings. Medicinal plants and their constituents increase the anti‐cancer effects of many chemotherapeutic agents and radiotherapy, as well as reverse the effects of chemotherapeutic drug resistance, which is noteworthy since the emergence of chemotherapeutic drug resistance will eventually compromise the treatment of cancer.

The reviewed papers showed that the extract of *Z. multiflora* extract and its main constituent carvacrol, reduce lung cancer virality, increases the expression of apoptotic proteins, modulates cytokine levels, cancer cell viability and regulate specific pathways. The effect of *C. sativus*, crocin and crocetin on down‐regulation of mRNA levels of major proteins of apoptosis in lung cancer cells was also reported. *F. persica* and its main constituent coumarin are shown as a possible treatment for lung cancer via the inhibition of cyclins and CDK, their reducing effects on cancer cell migration and metastasis mediated by cytokines such as IL‐1β, oxidative marker production, cell viability and cytotoxicity.

*C. longa* and CUR suppress cancer cell proliferation and induce apoptosis of lung cancer cells. CUR changed the expression of EGFR, microRNAs and autophagy in cancer stem cells and influences the expression of different genes in the cancer cells, such as NF‐kB, STAT‐3 and AP‐1, as well as protein kinases, including MAPK and enzymes such as COX and LOX. The anti‐cancer mechanisms of *A. millefolium* and its constituent, kaempferol by change in cancer cell immobility and death as well as disruption the cytoskeleton of lung cancer cells were shown. *P. oleracea* and its constituent such genestein reduce cell viability, cell proliferation, Bcl‐2 level and MMP‐2 expression but increase apoptotic, cytotoxicity, Bax expression, miR‐128 activation and p21 protein level, inhibit NSCLC cells ERK1/2 and PI3K/Akt phosphorylation, regulate Cas‐3/9 activity, miRNA27a activity and the expression of the MET protein enhancing p62, activating Cas‐3 and Cas‐8 and increasing TRAIL‐induced tumour cell death by activated autophagy.

The anti‐cancer effects of *A. cepa* and its main constituent QT are shown by adenocarcinomas parameters inhibition through modulating the expression of B‐cell lymphoma, Bax mRNA, Bcl‐2 mRNA, cell viability and DNA synthesis in lung cancer cells. The effects of *B. juncea* or mustard and its constituents on lung cancer treatment are shown via increasing antioxidant activities and antiproliferation activity markers, apoptosis and improving levels of immunoglobulins, Cas, HMGB1, LDH and pro‐inflammatory cytokines. Based on the above studies, *A. vera* and *A. emodin* showed anti‐carcinoma effects on lung cancer cells. It was shown that *A. vera* and *A. emodin* are effective in lung cancer therapy by inhibiting cell proliferation, cytoskeleton activation, stimulated cell apoptosis as well as Cas‐3 and PKC activation, MAPK signalling, inhibition of ROS production, Akt/mTOR and PI3K/AKT pathways.

Therefore, promising effects of medicinal plants and their constituents on lung cancer based on many experimental findings were indicated. However, it is necessary to pay attention to the pharmacodynamic and pharmacokinetic limitations of each medicinal plant. In addition, further clinical studies of the plants and their constituents on lung cancer are needed to justify their use in clinical practice for the treatment of these diseases.

## AUTHOR CONTRIBUTIONS

**Arghavan Memarzia:** Writing – original draft (equal). **Saeideh Saadat:** Writing – original draft (equal); writing – review and editing (equal). **Fereshteh Asgharzadeh:** Writing – original draft (equal); writing – review and editing (equal). **Sepide Behrouz:** Writing – original draft (equal). **Gert Folkerts:** Writing – review and editing (equal). **Mohammad Hossein Boskabady:** Formal analysis (equal); methodology (equal); supervision (equal); writing – review and editing (equal).

## FUNDING INFORMATION

Not applicable.

## CONFLICT OF INTEREST STATEMENT

The authors declare that they have no competing interests.

## CONSENT TO PARTICIPATE

Not applicable.

## CONSENT TO PUBLISH

Not applicable.

## Data Availability

Not applicable (This is a review article).
